# Supplementary material for: Are carbon emissions trading and green financial instruments synergistic? -Comprehensive quantitative research based on content analysis
Source: PLoS One. 2024 Mar 7;19(3):e0298601. doi: 10.1371/journal.pone.0298601 (PMC10919601; doi:10.1371/journal.pone.0298601)
Supplement: S1 Data — (ZIP) [file pone.0298601.s001.zip › Data/地级市Z_F工作报告.zip!/░▓╗╒/┬φ░░╔╜╩╨/└┤╫╘╣1⁄2╓┌║┼í╛┬φ┐╦╩2╛▌═°í┐_2006.docx]

# **政府工作报告（2006）**

【字体大小：[大](http://zwgk.mas.gov.cn/openness/detail/content/javascript:doZoom(18)) [中](http://zwgk.mas.gov.cn/openness/detail/content/javascript:doZoom(15)) [小](http://zwgk.mas.gov.cn/openness/detail/content/javascript:doZoom(14))】

——2006年1月10日在马鞍山市第十三届人民代表大会第五次会议上

市  长   姚玉舟

各位代表：

现在，我代表市人民政府向大会作工作报告，请予审议，并请市政协委员和其他列席人员提出意见。一、2005年工作回顾及“十五”主要成就

刚刚过去的2005年，是我市推进东向发展、加速“两个率先”、构建和谐社会取得显著成绩的一年。一年来，在省委省政府和市委的领导下，我们认真贯彻党的十六大和十六届四中、五中全会精神，全面落实科学发展观，积极创新工作思路，勇于破解各种难题，保持了经济社会发展的强劲势头，圆满完成了市十三届人大四次会议确定的目标任务。预计2005年全市实现生产总值325亿元，比上年增长19%，人均生产总值迈上3000美元台阶，达3200美元；完成全社会固定资产投资190亿元，增长30.6%；财政收入突破60亿元，增长31%；实现社会消费品零售总额64.3亿元，增长12.6%；城市居民人均可支配收入1.2万元，农民人均纯收入4400元，分别增长17.8%和10.4%；年末城镇登记失业率为3.7%；人口出生率控制在10.5‰。在过去一年中，我们成功承办了第一届中国诗歌节，顺利通过创建国家环保模范城市考核验收，获准建设国家金属矿产资源高效循环利用工程研究中心，再次获得“全国科技进步先进市”荣誉，被授予“全国民族团结进步模范市”、“全国按比例安排残疾人就业先进城市”称号，经济普查工作、人防工作分别受到国务院和国家国防动员委员会表彰，华菱集团进入中国自主创新品牌60家最具成长力企业行列，马钢牌热轧H型钢成为“中国名牌产品”，实现我市中国名牌零的突破。

一年来，市政府重点开展了八个方面的工作：

（一）以加快工业发展为重点，“三大基地”建设取得新突破。一是强力推进现代加工制造业基地建设。加大工业有效投入，全年完成工业投资首次突破100亿元。马钢500万吨薄板带项目快速推进，蒙牛冰淇淋一期、海螺水泥粉磨站等41个重点工业项目建成发挥效益，万能达二期扩建、山鹰造纸等53个项目正在加紧建设，工业经济发展后劲进一步增强。出台了促进中小企业发展的政策措施，启动实施“万千百十”工程，积极推动银政银企合作，省工行、农行、建行和市商行累计授信210亿元，其中支持中小企业发展的额度达80亿元。全面推进国家863新材料基地主体园区、慈湖新材料产业园及当涂新材料产业园建设，佳达软件园、华骐环保科技园、菲利特新型水处理环保设备等一批高科技项目入园发展，全市高新技术产业产值突破100亿元。深入开展质量兴市工作，积极实施名牌发展战略，海狮牌毛巾等6个产品跻身“安徽名牌产品”行列，洪滨丝画荣获第54届尤里卡世界发明博览会“特别金奖”。加强工业经济运行服务，千方百计化解能源、资金等要素制约，促进工业经济平稳较快增长。全年实现规模以上工业增加值198亿元，增长30.7%；工业经济综合效益指数达235；新增规模以上工业企业77户，总数达341户，销售收入超亿元企业达29户，其中超10亿元企业8户。马钢产能跃上1000万吨新台阶，实现销售收入330亿元，向具有国际竞争力的现代化企业集团目标迈出坚实步伐。二是加快推进绿色食品基地建设。在稳定粮食种植面积基础上，不断扩大水产生态养殖和蔬菜园艺产品生产，品质和效益继续提升。支持农业产业化龙头企业加快发展，全年新增省级龙头企业2户，安民农副产品批发市场年交易额突破4亿元。推进农业标准化生产，17个农产品获得绿色食品认证，1户企业通过无公害农产品产地认证。实施“农产品进超市”工程，优质农产品销售渠道进一步拓宽。贤进牌河蟹行销东南亚和港澳台地区，实现我市农产品自营出口创汇零的突破。加大“三资”农业发展力度，全年新建、扩建“三资”农业项目172个，到位资金4.5亿元，蒙牛现代牧场开工建设。全年粮食总产量达38.5万吨，完成农业增加值17.5亿元，保持较好发展水平。三是扎实推进休闲旅游基地建设。以采石风景区建设为重点，完成锁溪河环境综合整治一期工程，开工建设翠螺山休闲文化园。坚持生态优先，着手制定实施濮塘地区总体规划，着力引进项目打造独具特色的旅游区。大力创新旅游宣传促销方式，深化区域旅游合作，全年实现旅游业总收入11.8亿元。继续加强商业网点规划和建设，建成市中心商业步行街，大华购物中心一期工程建设快速推进，新都商品批发市场、沃尔玛购物中心、海外海广场等项目启动实施，解放路等特色商业街进一步繁荣，全市商贸服务功能明显提升。金融业保持稳健运行态势，支持经济发展力度加大，年末本外币存贷款余额分别比年初增加44亿元和18亿元。

（二）抢抓机遇加快东向发展，对外开放水平全面提升。认真贯彻省委省政府东向发展重大决策，落实省委加快毗邻苏浙地区和沿江四市发展座谈会精神，制定强势推进东向发展、加速实现“两个率先”的意见，明确了当好安徽奋力崛起脊梁和东向发展排头兵的目标措施，在规划、交通、市场、人才等方面与南京都市圈和长三角城市开展了卓有成效的合作。密切跟踪国际国内产业和资本转移新动向，主动出击长三角等发达城市和日本、韩国、俄罗斯、西班牙等国家及地区，全方位开展招商引资。成功举办诗歌节经贸洽谈会，组团参加徽商大会、南京重洽会、杭州经济合作推介会、厦门投资贸易洽谈会、吉林·东北亚投资贸易博览会、日本爱知世博会等重点招商活动，加入了“东北亚机械工业城市联合体”，促成马钢BOC工业气体、中橡扩能、圣戈班配套、雨润肉制品等一批重大项目签约。切实加强招商引资调度，建立重点项目工作责任制，全年实际利用外资1.5亿美元、内资45亿元，分别增长36.4%和18.1%，呈现出总量快速增长、单个项目投资规模增大、投资领域不断拓宽的新特点。大力推进开发园区建设和发展。编制完成马鞍山经济开发区南区总体规划，污水处理厂一期工程建成投入运行，区内道路等基础设施进一步完善，当年实际利用外资9000万美元，实现技工贸收入60亿元，财政收入突破2亿元，主要经济指标连续三年位居全省省级开发区首位，正式申报国家级开发区。慈湖经济开发区进入省级开发区行列，在建项目18个，完成固定资产投资15亿元，实际利用外资2050万美元、内资7.5亿元，实现规模以上工业增加值6.4亿元。鼓励和引导企业积极开拓国际市场，共完成外贸进出口总额11.5亿美元，增长33.6%，其中出口2.5亿美元，增长43.7%，全市经济外向度提高到28.7%。加快“走出去”步伐，十七冶、中冶华天等企业对外工程承包和技术输出稳步发展，全市外经营业额达500万美元，增长86.1%。与芜湖市在规划、人才、科研等方面开展合作，推动皖江区域联动发展取得实质性进展。

（三）进一步深化各项改革，体制机制不断创新。深入推进企业改革工作，顺利完成金星化工、港口集团、纺织品总公司和马钢医院改制，引资盘活原市橡胶厂、皖通公司、合力公司闲置资产，全市工业企业改革全面完成；积极开展企业改制“回头看”，突出抓好提留资产监管等工作，巩固和发展了中小企业改革成果。启动生产经营类事业单位改革，完成转企改制38户。积极推行聘用制和人事代理，教育、卫生系统人事制度改革迈出新步伐。实施新一轮市及县区政府机构改革，促进了政府职能转变。扎实开展农村综合改革试点，乡镇机构改革、县乡财政体制改革、农村义务教育管理体制改革和建立农村基层工作新机制有序推进。金融改革进一步深化，在全国地级市中率先组建农村合作银行，市商业银行成功参与徽商银行联合重组。认真贯彻国务院和省政府加快发展非公有制经济的政策措施，深入实施创业扶持工程，支持工会、共青团、妇联组织实施“职工经济技术创新工程”、“新世纪帮助青年创业行动”和“巾帼创业行动”。继续强化城区创业服务中心功能，改革企业和个体工商户登记管理办法，进一步优化了民营经济发展环境。成功举办第二届中国非公有制经济发展论坛马鞍山分会场活动，促进了与全国知名民营企业的交流和合作。全年新增个体工商户6700多户，新增民营企业多户，个体私营经济纳税额突破5亿元，占地方财政收入的比重达18%，有力地促进了全市经济社会发展。

（四）强化城市建设和管理，创建国家环保模范城市工作通过考核验收。大力推进城市规划、建设和管理工作。邀请国内外专家参与城市规划编制，着力提高城市规划水平。继续加快推进城市基础设施建设，全年完成城建投资41.2亿元。九华路三期、慈湖河路二期等10条新建道路基本建成，205国道一期、长江路等5条道路改造全面完工，新增城市道路总里程17.3公里。马芜高速公路建成通车，长江大桥项目前期工作取得重大进展。加强电力设施建设，江东变电所竣工投入使用，太白开关站等电力工程进展顺利。港口建设快速推进，马鞍山港成为全国内河主要港口之一，年货物吞吐量超过2000万吨。加快发展公用事业，第四水厂建成试运行，向山生活垃圾无害化处理一期工程竣工投入使用，王家山污水处理厂改扩建工程开工建设。认真贯彻房地产宏观调控政策，推动房地产市场健康发展，全年完成房地产开发投资26亿元，竣工房屋面积104.5万平方米。归集住房公积金5.3亿元，促进了职工住房消费。加强城市绿化美化工作，葛羊路城市入口广场绿化改造基本完成，采石河综合整治一期工程按计划推进。依法加强城市管理，继续规范相对集中行政处罚权制度，加大城乡结合部环境整治力度，推行散装货物运输车辆密闭改装，进一步规范户外广告设置。制定实施《马鞍山生态市建设总体规划》，生态市建设迈出可喜步伐。积极发展循环经济，广泛推行清洁生产，节能降耗工作取得新进展，全市单位生产总值能耗达到全国同类城市先进水平，工业固废处置率和利用率分别达到80%和60%。扎实推进矿山整治，着力抓好炉窑烟尘治理，深入开展查处环境违法行为专项行动，全年空气质量优良率达90%以上。经过全市人民共同努力，创模工作在安徽省、中部地区及全国钢铁工业城市中第一个通过国家环保总局考核验收。

（五）加大扶持引导力度，县区发展活力明显增强。支持县区加快各类园区建设。当涂经济开发区被批准为省级开发区，累计入区项目44个，32个项目建成投产。雨山、金家庄两个工业园有20个项目建成发挥效益，花山工业集中区基础设施建设步伐加快，博望等一批乡镇工业集中区的集聚效应日益显现。按照统筹城乡发展的要求，启动新一轮村镇规划编制工作，乡镇总体规划修编结束，村庄布点规划基本完成。加快农村交通设施建设，围乌、新黄、澄湖等公路建设按计划推进，有200多公里的部省通达工程竣工通车，总投资2亿元的当涂县村村通水泥路工程进展顺利，市郊村村通工程开始实施。小城镇建设完成投资6.1亿元，中心镇服务功能明显增强。在落实免征农业税等惠农政策的基础上，制定出台了进一步支持“三农”的17条措施。按政策提高粮食补贴标准，兑现各类补贴资金近3000万元，调动了广大农民的生产积极性。安排专项资金6100多万元，支持农业产业化、水利交通基础设施和农村安全饮水工程建设，改善了农民生产生活条件。转移支付4500多万元，保障了乡镇政权、村级组织的正常运转。统筹城乡就业培训，全年培训进城务工农民和被征地农民4357人。强化农民劳务输出的组织和管理，全年新增劳务输出2.2万人。积极推进万亩以上圩堤加固达标和水系治理，建成黄池排涝二站和石臼湖堤防护坡应急工程。继续实施病险水库和涵闸除险加固，推进丘陵区当家塘建设，城乡防洪保安和抗旱减灾能力得到新提高。去年，当涂县财政收入突破5亿元，三区财政收入增幅均在35%以上，县区经济呈现蓬勃发展态势。与此同时，县区政府努力做好土地征迁和农民安置工作，为保障全市经济快速发展作出了积极贡献。

（六）努力解决事关群众切身利益的问题，人民生活继续改善。认真落实各项就业扶持政策，广泛开辟就业渠道，加大就业培训力度，实行“一对一”职业介绍服务，全年新增就业2.7万人，其中失业人员再就业1.8万人，“4050”人员再就业3428人。继续完善社会保障制度。推行社会保险费统一登记、一票征收，城镇职工养老、失业、医疗、工伤、生育保险基金征缴率均在95%以上。马钢等企业成功实施年金制度，多层次的职工养老保险体系正在形成。全面推行新型农村合作医疗制度，当涂县和市郊农民参保率分别达到92.5%和94.4%。进一步完善城镇低保制度，实施市郊农村低保提标扩面，启动当涂县农村低保试点。积极推进企业退休人员社会化管理，社区管理服务率提升至78%。实施建设领域农民工工资支付保障制度，切实维护劳动者合法权益。建立城乡特困群体医疗保障制度，启动实施城区低保对象重病救助和农村特困户、五保户大病救助，困难职工帮扶救助机制逐步完善。实施城乡特困家庭子女入学援助制度，对九年义务教育阶段特困生免除杂费，并对农村特困生免费提供教科书，对高中阶段特困生免除30%学费。出台了特困家庭大中专毕业生就业援助办法。切实保护被征地农民和城市拆迁户合法权益，各类征迁补偿款及时足额到位；全面实施被征地农民基本养老保障制度，8808名参保农民开始领取养老保障金；被征地农民安置房加速建设并部分交付使用。雨山湖公园、朱然墓园免费向市民开放。农村安全饮水工程按计划推进，农村卫生厕所普及率提高到60%。去年11件为民办实事项目，除2件跨年度实施外，其余均如期完成。

（七）协调发展科技教育和各项社会事业，城乡文明程度得到新的提高。加快科技创新和科技成果转化步伐。矿院联合马钢、安工大等单位组织申报国家金属矿产资源高效循环利用工程研究中心获准建设。天源科技公司被认定为国家火炬计划重点高新技术企业。科技创业服务中心在孵企业产值突破1亿元，后续工程正在加紧建设。深化产学研合作，成功举办国际高新技术转移合作研讨会，企业与高等院校和科研院所的合作迈上新台阶。全年专利申请量达265件，增长40%。大力发展教育事业。开展城市示范学校与农村薄弱中小学合作办学，实施第二轮中小学布局调整，促进了义务教育的均衡发展。放大优质教育资源效应，省、市示范高中招生规模进一步扩大。市工业学校被批准为国家级重点中等职业学校，市七中被命名为全省首批特色初中，安徽工贸技师学院竣工投入使用并通过省政府评估验收。启动建设留学人员创业园，加快构筑高层次人才创业载体。积极发展文化、卫生、体育事业。成功承办第一届中国诗歌节，进一步挖掘了马鞍山人文底蕴，全面展示了我市改革发展成果和对外开放的良好形象。城市语言文字工作达到国家二类城市标准。市疾病预防控制中心和紧急救援中心投入使用，市传染病医院加紧建设，公共卫生基础设施进一步完善，重大疾病预防工作取得显著成效。食品药品放心工程全面实施，食品质量安全市场准入制度正式建立。群众性体育活动蓬勃开展，成功举行第三届社区运动会，我市运动员在全国十运会上夺得3金2银1铜，为马鞍山和安徽省争得了荣誉。启动实施农村部分计划生育家庭奖励扶助制度，出生人口性别比综合治理工作扎实开展。数字电视节目平台正在加速建设，农村有线电视“村村通”工程稳步推进。基层民主政治建设得到加强，完成第六届村委会换届。高质量完成第一次经济普查工作，摸清了全市经济家底。其他各项社会事业都有新的发展。

全面启动“平安马鞍山”创建工作。积极构建“点线面”相结合的动态防控体系，重拳开展“打黑除恶”、侦破命案、扫除“黄赌毒”等专项行动，社会治安形势良好。建立信访联席会议制度，严格落实信访工作责任制、重点信访案件领导包案制，开展领导干部开门接访、带案下访活动，解决群众关心的热难点问题，有效化解社会不稳定因素。认真落实安全生产责任制，加大事故隐患清查整治力度，着力减少各类安全事故。高度重视社会预警体系建设，完善突发事件应急预案，政府防范和应对公共危机的能力明显增强。强化公民道德教育，深入推进“信用马鞍山”建设，积极开展文明城市创建活动，大力弘扬马钢创业精神，城市文明程度和市民文明素质得到新的提高。加强学习型城市建设，完成与清华大学共建学习型城市示范工程课题验收。广泛开展军地军民共建活动，海军“马鞍山舰”正式命名，创建全国双拥模范城市工作扎实推进，再次获得省“双拥模范城”称号。在去年“8·2”火灾战斗中，公安消防官兵奋不顾身，全力扑救，最大程度地保障了人民群众生命财产安全。郑飞、管志彦、叶晓辉三名战士用青春和生命，书写了光辉的人生篇章，被推选为2005年度“中国骄傲”人物，在人民群众心目中树起一座永恒的丰碑！

（八）切实履行政府职责，行政能力建设进一步加强。全市各级政府扎实开展以实践“三个代表”重要思想为主要内容的保持共产党员先进性教育活动。市政府领导班子结合实际开展了“有理想、有责任、有能力，好作风、好业绩、好形象”主题实践活动，紧紧抓住取得实效这个关键，落实了9个整改专题、68项整改任务，取得了促进各项工作的明显成效。一年来，市政府坚持向市人大报告工作、向市政协通报情况制度，自觉接受市人大法律监督、工作监督和市政协民主监督。制定并实施了市政府领导领衔办理人大代表议案、政协提案制度，市政府领导班子成员定期约请和走访、看望市人大代表、市政协委员，听取对政府工作的意见和建议。政府系统承办的10件人大代表议案（并案为3件）、129件建议和383件政协提案如期办复。继续完善重大决策的规则和程序，广泛集中民智，推进决策的科学化、民主化。深入实施行政审批制度改革，进一步规范行政行为。完成“四五”普法扫尾任务，依法治市工作成效显著。按照教育、制度、监督并重的要求，加强政府系统廉政建设。制定了强化财政性投资项目管理的具体措施，开展政府性投资项目和农民安置房建设专项执法监察。强化本级预算执行审计，首次实行审计结果公告。出台加强行政机关效能建设的规定，积极发挥为民办实事义务监督员的监督作用，开通政风行风热线，深入推行政务公开，促进了“阳光政府”建设。

2005年全市经济社会发展取得的显著成绩，为“十五”计划的全面超额完成画上了圆满的句号。过去的五年，是新世纪新阶段新发展的五年，也是硕果累累令人自豪的五年。这期间，我们积极适应我国加入世贸组织和中央实施宏观调控带来的新变化，沉着应对要素资源约束加剧的严峻考验，妥善化解非典威胁、高致病性禽流感等重大疫情造成的不利影响，在抢抓机遇中加快发展，在整体推进中寻求突破，推动改革开放和现代化建设不断迈上新台阶，为“十一五”更快更好更全面发展奠定了坚实基础。

“十五”时期是全市经济实力提升最快的时期。与“九五”末相比，全市生产总值翻了一番多，年均增速高达14.8%，高出预期目标5.8个百分点，总量由全省第12位跃居前6位。规模以上工业增加值实现翻两番，总量由全省第3位跃居第2位，对全省的贡献率达19%，综合经济效益指数比“九五”末提高143个百分点。全市工业化水平提高15个百分点，达到66.2%。全社会固定资产投资累计完成539亿元，年均增速达44.1%，投资额连续三年位居全省前3位。工业性投资比重过半，其中马钢投资150亿元实施了以冷热轧薄板为龙头的一批重大技改项目，有力促进了产品结构的优化升级和市场竞争力的提升。社会消费品零售总额累计完成247.9亿元，年均增速为12.6%。财政收入连跨20—60亿元五个台阶，年均增速达26.6%，总量由全省第7位跃升为第3位。县区经济发展实现新跨越，当涂县财政收入增长3.3倍，三个区财政收入全部翻了两番以上。全市经济实力大幅提升，跻身中国综合实力百强城市行列。在主要经济指标人均值继续以较大幅度保持全省领先的同时，多数主要经济指标增幅超过长三角城市平均水平。

“十五”时期是全市对外开放成效最大的时期。开发园区建设取得重大突破，项目建成区面积达8平方公里，成为拉动经济增长的重要力量。利用内外资总量大幅攀升，全市累计实际利用外资3.9亿美元、实际利用内资123.4亿元，分别是“九五”时期的5.4倍和6.2倍。外经外贸工作深入拓展，外贸进出口总额连续三年排名全省第二，五年累计完成34.7亿美元，年均增长36%，其中出口完成7.7亿美元，年均增长17.1%。对外友好交流与合作稳步推进，与西班牙阿尔冈达·德雷伊市、澳大利亚科克拉市和陕西省宝鸡市缔结为友好城市，并获得外事审批权。区域经济合作取得丰硕成果，融入长三角经济圈、参与南京都市圈建设和共同打造皖江现代化城市群工作取得实质性进展，全方位、多层次、宽领域的对外开放格局基本形成。

“十五”时期是全市改革创新力度最强的时期。按照“三个置换、两个结合、一个保障”的总要求，坚持因企制宜、规范操作，全市共完成国有、集体中小企业改革404户，涉及职工9.2万人，支付改革成本20.6亿元，其中通过债务重组收益10亿多元，提前两年完成省政府下达的中小企业改革任务。骨干企业上市融资实现新突破，山鹰、星马公司先后成功上市，山鹰公司两次实现再融资。马钢、十七冶等企业主辅分离工作取得重要进展，企业办社会职能实现平稳移交。矿院、设计院成功实行企业化改制，在参与地方经济建设中发挥了重要作用。城市公用事业单位改革成效显著，自来水、燃气、公交公司引进外资完成全面改制，生产经营类事业单位转企改制取得重要突破。农村改革稳步推进，在全省率先免征农业税及其附加，农村税费改革成果得到巩固。行政管理体制改革不断深入，调整优化行政区划布局，实施对区放权，建立了马鞍山经济开发区财政管理体制，激发了县区和马鞍山经济开发区自主创业发展的能力和活力。按照上级部署，两次完成市、县（区）政府机构改革任务。缩减行政审批项目，完善行政服务中心功能，强化行政效能建设，政府职能加快转变。民营经济发展势头强劲，增加值年均增长24.6%，对全市经济增长的贡献率达41.9%，其中民营企业近三年每年新增1000户以上，纳税额年均增长45.4%。

“十五”时期是全市城乡面貌变化最显著的时期。确立了建设“双百”现代化城市的战略目标，完成新一轮城市总体规划和综合交通、商业网点等专项规划编制工作，城镇总体规划体系进一步完善。大规模展开城市建设，五年累计完成城建投资146.6亿元，是“九五”时期的3.1倍，市县建成区面积扩大到80平方公里，“双百”城市构架初步拉开。城市道路建设完成投资23.7亿元，先后建成江东大道等一批城市主干道，新增城市道路总里程81公里；扩建313、314省道和马濮旅游大道，开辟了至禄口机场的新通道，新增农村道路840公里，城乡交通条件得到显著改善。房地产开发投资完成76.9亿元，竣工房屋面积417.6万平方米，是“九五”时期的2.2倍。小城镇建设快速推进，累计完成投资21.3亿元。“数字马鞍山”建设取得新成果，公共信息服务水平不断提高。城市公用设施日趋完善，长江干堤加固达标等重点水利工程顺利实施。着力打造绿色马鞍山，建成区绿化覆盖率提高到42%，人均绿地面积达11.8平方米。雨山湖综合整治、南排工程和雨山、佳山公园建设等一批重点项目的实施，进一步改善了城区内的环境。基本农田保护措施得到加强，国土资源管理工作取得新成绩，可持续发展能力不断增强。

“十五”时期是全市人民群众得到实惠最多的时期。通过发展经济、落实政策、加强培训和扶持创业等措施，着力解决城乡居民的就业再就业问题，五年新增就业岗位9.7万个，4.7万名下岗失业人员实现再就业，新增农村劳务输出5万人。城乡居民生活水平大幅提高，期末城市居民人均可支配收入、农民人均纯收入分别比“九五”末净增5096元和1717元，年均增长11.7%和10.4%；城乡居民人均储蓄由“九五”末的5673元增加到1.4万元；城镇职工住房公积金归集额由“九五”末的7.6亿元增加到25亿元；城镇居民住房建筑面积比“九五”末净增3.8平方米；汽车进入部分居民家庭。以养老、失业、医疗、工伤、生育五大保险和城市低保为主要内容的社会保障体系逐步完善，社会救助体系框架初步建立，基本实现老有所养、病有所医、困有所济、有房可住、有事可做、有学可上的社会保障目标。五年共实施为民办实事项目49件，解决了与群众生产生活密切相关的一批热点、难点问题。在最新公布的“中国城市生活质量指数”百强排行榜上，我市综合排名列第78位。与此同时，高度重视社会治安综合治理工作，积极推进“平安马鞍山”建设，妥善处理改革发展稳定的关系，统筹抓好民主法制建设、精神文明建设和社会事业发展，促进了经济、政治、文化和社会建设“四位一体”协调发展。

在过去五年的实践中，我们始终坚持发展为上，抢抓重要战略机遇期，把加快发展作为最大的政治、最硬的道理、最根本的任务，苦干实干，攻坚克难，奋力取得经济社会发展的重大突破；始终坚持从发展大局出发，保持政府工作的连续性和稳定性，全力组织实施招商引资、改革攻坚、重点建设、农村发展、就业和社会保障等重点工作，推动“十五”各项目标任务全面超额完成；始终坚持抓早抓实，超前谋划，做到早安排、早启动，努力把形势研究得更透，把政策领会得更深，把决策制定得更科学，争取工作的主动权；始终坚持以解放思想为先导，自觉破除一切不合时宜的落后观念，勇于面对困难，敢于承担责任，变挑战为机遇，变压力为动力，凝心聚力谋发展，逐步走上一条符合时代要求、具有自身特点的加快发展之路；始终坚持以人为本、富民为先，高度关注困难群体的生产生活，着力解决事关群众切身利益的突出问题，让改革发展的成果最大程度地惠及广大人民群众，努力实践全心全意为人民服务的根本宗旨。

各位代表，“十五”成就来之不易，令人鼓舞。这是在中央路线方针政策的指引下，按照省委省政府的决策部署，在市委的坚强领导下，在市人大、市政协的监督帮助下，在社会各界的大力支持下，全市各级政府依靠广大人民群众共同奋斗的结果。在此，我代表市人民政府，向辛勤工作在全市各条战线上的广大干部群众，向市人大代表、市政协委员，向各民主党派、工商联、人民团体和社会各界人士，向中央和省驻马单位、来马投资企业，向驻马解放军、武警官兵和公安民警，表示衷心的感谢并致以崇高的敬意！

回顾过去的五年，我们清醒地看到，前进的道路上还存在不少困难和问题，主要表现在：一是经济结构性矛盾仍然比较突出，县区经济所占份额不大，民营经济实力较弱，服务业发展相对滞后，经济外向度不高。特别是规模以上企业数量少，产品结构较为单一，经济抗风险能力有待进一步增强。二是资源环境约束加剧，土地供应日趋紧张，能耗水耗仍然较高，实现可持续发展的压力较大；人才结构和分布不合理，高层次企业管理人才、高技能型人才明显不足，难以适应经济社会发展的要求。三是对照和谐社会的要求，就业压力依然较大，农村社会保障体系还不完善，农民持续增收的基础还不稳固，少数城乡群众生活还比较困难，维护社会稳定还有大量工作要做。四是城市管理水平有待提高，文明创建、社会治安综合治理的长效机制亟待健全。五是政府职能转变还不适应经济和社会转型的要求，政府一些工作人员特别是少数干部思想不解放、精力不集中、工作不落实、服务不到位的现象还不同程度存在。对这些问题和不足，我们已经并将继续通过加快发展和深化改革等措施逐步加以解决。

二、“十一五”发展的指导思想和目标任务

“十一五”时期，我市面临着新的发展机遇和挑战。从机遇看，经济全球化和区域经济一体化深入发展，产业升级和梯度转移步伐进一步加快，沿海发达地区特别是长三角地区产业和资本加速向周边及中西部转移，为我市发挥比较优势、承接产业和资本梯度转移，创造了有利的条件。特别是中央作出促进中部地区崛起的重大决策，省委省政府推进东向发展，把马芜铜宜列为第一方阵，要求当好安徽崛起的脊梁和排头兵，这些都为我市争取政策支持、加快结构调整、转变增长方式、实现更大发展，提供了难得的机遇。从挑战看，随着一些行业产能加速释放，部分产品供过于求的矛盾已经显现，尤其是钢材等重要投资品出现了全国性产能过剩问题。我市以加工制造业特别是钢铁工业为主的产业结构，正面临着市场竞争日趋激烈、资源和环境约束不断加剧、结构调整任务艰巨的严峻考验。同时，统筹经济社会协调发展的任务繁重。总之，“十一五”时期，我们面临的仍将是一个机遇和挑战并存、机遇大于挑战的环境，一个总体上有利于促进经济社会发展、但不利因素可能增多的环境。我们一定要进一步认清形势，增强忧患意识和责任感，未雨绸缪，趋利避害，努力开创改革开放和现代化建设新局面。

根据《中共马鞍山市委关于制定马鞍山市国民经济和社会发展第十一个五年规划的建议》，“十一五”时期全市经济社会发展的指导思想是：坚持以邓小平理论和“三个代表”重要思想为指导，认真贯彻中央和省委省政府各项决策部署，以科学发展观统领经济社会发展全局，积极贯彻东向发展战略，大力推进工业化、城市化、市场化、国际化进程，全面融入长三角发达地区，加速调整经济结构，转变经济增长方式，提高自主创新能力，推动经济超常规、跨越式发展，加快推进现代加工制造业基地、绿色食品基地、休闲旅游基地建设，促进社会主义经济、政治、文化和社会建设协调发展，全力推进全民创业、富民强市，加速实现“两个率先”，努力构建和谐社会，为实现安徽奋力崛起作出新的更大贡献。

“十一五”时期全市经济社会发展的主要目标是：在优化结构、提高效益和降低消耗的基础上，到规划期末，实现经济总量比“十五”末翻一番，达650亿元，力争实现700亿元，人均生产总值比2000年翻两番，达6000美元，力争达到6500美元；财政收入与生产总值保持同步增长，达140亿元；城市居民人均可支配收入达到17000元以上，农民人均纯收入达到7100元以上，年均增幅分别达7.2%和10%；主要总量指标进入全省最前列，占全省份额进一步提升，人均主要经济指标以较大优势在全省领先，达到长三角城市中等发展水平；资源利用效率显著提高，单位生产总值能耗持续下降，保持全国同类城市先进水平，到2010年单位生产总值能耗比“十五”末降低18%，力争降低20%；改革开放取得新的重大突破，社会主义市场经济体制不断完善，率先融入长三角，加速实现国际化，开放型经济水平迈上新台阶；经济结构调整取得重要进展，形成一批拥有自主知识产权和知名品牌、具有较强竞争力的优势企业和产业集群；城乡统筹协调发展的大格局基本形成，城市化水平得到新提高，社会主义新农村建设取得积极进展；环境保护和生态城市建设取得新进步，资源节约型和环境友好型社会框架初步形成；城乡人民生活质量显著提高，城镇就业岗位持续增加，社会保障体系更加健全，基本普及高中阶段教育，基本医疗保障实现全覆盖，居住、交通、文化等方面条件进一步改善；精神文明建设和民主法制建设扎实向前推进，社会治安和安全生产状况进一步好转，构建和谐社会取得新进展。

“十一五”时期全市经济社会发展的主要任务是：

——全面深入实施大开放主战略。强力推进东向发展，重点开展与长三角的全方位对接，积极参与南京都市圈建设，加强与皖江城市的分工协作，在合作发展中实现互利共赢。继续举全市之力推进马鞍山经济开发区建设和发展，进入全省各类开发区前三强。加快慈湖经济开发区、当涂经济开发区和各类工业集中区发展步伐，规划建设大桥开发区，争取设立国家级高新技术开发区，构建开放型经济发展新载体。着力开展招商引资，不断优化投资环境，确保五年实际利用外资10亿美元以上、内资300亿元以上。实施对外贸易市场多元化、科技兴贸和出口商品品牌战略，力争外贸进出口总额年均增长15%。积??面提升对外开放水平。

——坚定不移地加快新型工业化进程。广泛应用高新技术和先进适用技术，改造提升传统产业，着力培育新兴产业，大力实施品牌战略，全力打造现代加工制造业基地。深入推进工业结构战略性调整。支持马钢做强钢铁主业，培育非钢支柱产业，早日成为具有国际竞争力的现代化企业集团；支持华菱集团增强产品研发和配套能力，加快建成全国重要的重卡和专用汽车生产基地；推动山鹰、长江钢厂、中橡、海螺、蒙牛等重点骨干企业快速发展。优化调整全市工业布局，突出发展特色产业集群，扩大优势产业规模效应。深入实施中小企业“万千百十”工程，促进“专精特新”企业结构优化升级，力争到2010年全市中小企业总数达1.5万户以上，其中规模以上工业企业1000户以上，年销售收入超亿元企业100户以上，新创省级以上名牌产品和著名商标10个以上。

——着力提高城市化水平。按照全面接轨长三角和打造皖江城市群的要求，进一步完善城市总体规划，加快编制城乡一体的城镇体系规划，切实发挥规划在城市建设和发展中的龙头作用。坚持以城市带动集镇，以集镇辐射农村，积极构建以市县主副城区为主、卫星镇和重点镇合理布局的城镇体系。推进主城区东扩、南进、西延、北接，市县建成区面积达到100平方公里左右。加快完善城市东部、南部路网和南接芜湖、北接江宁的基础设施，逐步形成畅通便捷的城市道路格局。适度超前建设城市公用设施，加强城市防洪保安体系建设，增强城市承载服务功能。建立健全城市长效管理机制，加大对突出问题的综合整治力度，不断提高城市管理水平。深化户籍、住房、就业、社会保障等制度改革，加速农村人口向城市转移，使城市化率每年提高2个百分点左右。

——扎实抓好社会主义新农村建设。按照“生产发展、生活宽裕、乡风文明、村容整洁、管理民主”的要求，高标准编制新一轮村镇规划，力争用5—10年时间完成村镇布点规划的实施，更好地促进工业向园区集中、人口向城镇集中、农业资源向能手集中。大力发展具有一定基础的乡镇特色产业集群和板块经济，整体提升农村工业化水平。加快现代农业建设，全面展开优势农产品生产布局，积极发展“三资”农业，推进农业产业化、标准化和规模化经营，打造面向长三角的绿色食品基地。强化政府对农村的公共服务，扩大公共财政覆盖农村的范围，统筹发展农村教育、文化、卫生等事业。加大农村基础设施和quo;、安全饮水等工程，全面展开大公圩等重要水系的综合治理。抓好城乡就业、社会保障等一体化建设，提高城乡统筹发展水平。扎实开展农村精神文明创建活动，培养有文化、懂技术、会经营的新型农民。

——推动服务业跨越式发展。加快服务业扩张和提升，力争2008年服务业增加值比2005年翻一番。积极培育发展物流产业，着力引进一批知名物流企业，加速构建多种运输方式相配套的物流运输网络。整合优质旅游资源，加快重点景区的深度开发建设，支持发展生态农业旅游和工业旅游，精心打造特色文化和休闲旅游品牌，初步建成与长三角互动发展的休闲旅游基地。继续保持房地产建设规模，改善供应结构，促进房地产业持续健康发展。努力提升金融生态环境建设水平，大力发展技术开发与服务、保险、会计、法律等现代服务业。同时，加快构建布局合理的市区两级商业服务网络，扩大钢材、装潢建材、绿色农产品等专业经营的容量，深入实施“万村千乡”农村市场工程建设，进一步提升传统服务业发展水平。

——大力推进重点项目建设。以大项目支撑大发展，突出抓好省“861”行动计划项目和市重点项目建设，为全市经济发展增添后劲。支持马钢、电厂、星马、山鹰等骨干企业加快实施扩建改造项目，促其尽快建成投产发挥效益。建成长江大桥及两端接线高速公路和城际客运轨道交通马鞍山段，改造宁芜铁路，加强长江及内河港口建设，积极推进芜申运河建设，进一步完善对外交通体系。密切跟踪国家产业政策导向，强化重大项目储备工作，建立项目储备机制，充实重大项目储备库。创新项目管理体制，推行政府投资项目代建制，确保项目建设的程序化、规范化、科学化。紧紧抓住中央促进中部地区崛起的重大机遇，积极争取国家和省里的政策及资金支持。加大资本运作力度，广泛开辟融资渠道，为重点项目建设提供资金保障。

——努力提升经济社会集约化发展程度。按照减量化、再利用、资源化原则，深入开展各类资源的循环利用，建设马钢、马鞍山经济开发区和慈湖经济开发区三大循环经济区，最大限度地提高资源利用效率。突出抓好重点耗能行业和重点企业的节能工作，千方百计降低资源消耗水平。推进国家清洁生产试点城市建设，建立节约型增长方式。积极开展废旧物资的回收利用，推进工业固体废弃物综合处置利用示范工程建设。加强矿山资源的综合利用和矿产资源的开采管理，实现规范化、保护性开采。强化全社会节约意识，倡导绿色消费，逐步形成与经济社会发展相适应的健康文明、节约资源的消费模式。认真落实生态市建设总体规划，扎实推进“生态马鞍山”建设，巩固和提高创建国家环保模范城市成果，努力建设资源节约型和环境友好型社会。

——坚持深化体制改革不动摇。深入推动思想解放，积极创新体制机制和政策措施，在全社会加快形成支持创新、扶持创业、激励创优的浓厚氛围。巩固完善企业改革成果，推动重点骨干企业深化改革，支持企业上市融资和上市公司再融资。基本完成事业单位改革任务，继续深化科技、教育、文化、卫生、体育等社会事业改革。推进财税管理体制改革，建立健全公共财政体系。深化投融资体制改革，规范政府投资行为。大力发展和完善产权、资本、技术、人才等要素市场，规范市场经济秩序，着力创造公开、公正、公平的市场环境。加快信用体系建设，建立健全守信激励与失信惩戒机制，建成全国信用最好的城市之一。强化政府社会管理和公共服务功能，继续扩大政务公开，加强行政效能建设，促进政府职能转变。深化城市基层管理体制改革，建设新型城市社区。

——加大科教兴市和人才强市战略实施力度。按照“自主创新、重点跨越、支撑发展、引领未来”的方针，大力推进国家“863”新材料基地建设，培育壮大高新技术产业。加快建设国家级和省级工程技术研究中心与重点实验室，支持马钢、华菱等企业自主创新项目进入省部共建范围，提升全市科技创新能力。突出抓好科技成果转化和应用，鼓励企业与高等院校和科研院所建立稳定的技术协作关系。全面推进“数字马鞍山”建设，初步形成覆盖全市的公共信息服务网络。加强科学技术普及，建设市科技馆。加快教育现代化步伐，全面实施素质教育，巩固提高义务教育水平，基本普及高中阶段教育，加快发展高等教育。大力发展职业教育、专业技能教育，努力建成全省和长三角地区职业教育基地。支持和引导民办教育持续健康发展，争取海内外资金投资、捐助教育事业。引导全社会树立全民学习、终身学习的理念，积极推进“学习型城市”建设。继续做好引进国外智力工作，加强高层次人才和紧缺急需人才队伍建设，优化人才发展环境，尽快形成人才辈出、人尽其才的生动局面。

——加快建设和谐马鞍山。继续实施积极的就业政策，以创业带动就业，以服务促进就业，突出抓好职业技能培训和就业创业服务，千方百计促进就业再就业。进一步完善城镇养老、失业、医疗、工伤、生育保险制度，推进农村最低生活保障制度和新型合作医疗制度建设，完善被征地农民养老保障制度，探索建立机关事业单位和农村基本养老保险制度，不断提高城乡社会保障水平。逐步推进全免费义务教育。积极发展慈善事业，扶助救济困难群体。完善城乡公共卫生和医疗服务体系，提高对重大疾病的预防控制和医疗救治能力。大力发展文化、体育事业，建成一批重大文体设施。进一步稳定低生育水平，提高出生人口素质，促进出生人口性别比趋向平衡。建立健全城乡居民收入稳定增长机制，扩大中等收入人群比重，提高低收入者收入水平。重视发展老龄事业，丰富老年人的精神文化生活。继续加强民族宗教工作。着力抓好安全生产各项措施落实，保持全市安全生产形势总体平稳。健全突发公共事件应急机制和社会动员机制，提高处置突发事件的能力。深入开展“平安马鞍山”建设，努力维护社会稳定。积极推进精神文明创建，继续加强民主法制建设，全面开展“五五”普法活动，提高依法治理工作水平。

三、扎实做好2006年各项工作，以更加优异的成绩迎接建市五十周年

2006年是实施“十一五”规划的开局之年，是我市加快实现“两个率先”第一个战略目标的关键之年，也是马鞍山建市五十周年的喜庆之年。在新的一年里，我们要以邓小平理论和“三个代表”重要思想为指导，认真贯彻党的十六大和十六届五中全会精神，坚持以科学发展观统领经济社会发展全局，积极应对经济运行中出现的矛盾和问题，突出抓好对外开放、全民创业、环境改善等重点工作，加快社会主义新农村建设，统筹发展社会事业，确保全面完成各项目标任务，为顺利实施“十一五”规划开好局、起好步。综合考虑各方面因素，今年全市经济社会发展的主要预期目标为：生产总值增长12%；财政收入增长12.5%；全社会固定资产投资增长22%以上；实际利用外资1.8亿美元，实际利用内资50亿元；外贸进出口增长14%，其中出口增长9%；社会消费品零售总额增长12%；城市居民人均可支配收入、农民人均纯收入分别增长10%；城镇登记失业率控制在4.5%以内；人口出生率控制在11‰以内；单位生产总值能耗下降4%左右。为此，我们将集中精力抓好以下九个方面的工作：

（一）着力实施大开放主战略，掀起招商引资新热潮

全力主攻招商引资。招商引资是现阶段推动经济加快发展的首要举措，也是抢占发展先机的重要途径。今年，要结合新一轮解放思想大讨论，在全市进行招商引资再发动、再聚力、再升温，掀起招商引资新热潮。围绕我市优势产业、优势资源、优势项目，充分发挥开发园区和重点骨干企业的主力军作用，着力引进科技含量高、产品附加值大、关联度强的大项目，提升引进外资的规模和质量。把长三角作为招商引资主阵地，建立覆盖苏浙沪地区的招商网络，加大力度组织小分队驻点招商，有针对性地组织策划重点招商活动，提高招商引资实效。进一步拓展招商引资领域，抓好现代服务业、农业的对外开放。强化对外来投资企业的跟踪服务，不断优化发展环境，吸引更多的外商来我市投资兴业。

强势推进开发园区建设。支持马鞍山经济开发区提速发展，确保全年实际利用外资1.2亿美元，工业产值增长50%以上。加快拓展马鞍山经济开发区发展空间，抓紧跨过采石河向南发展，基本完成南区“一纵三横”道路和配套基础设施建设，为项目入驻创造条件。着力完善慈湖经济开发区路网体系，加强与江宁滨江开发区基础设施衔接，大力发展循环经济，增强集聚辐射能力。积极推进新城东区基础设施建设，以高教园区为起步区，带动东部地区文化、生态旅游等相关产业发展。支持当涂经济开发区和各类工业集中区大发展，加强资源整合，促进功能提升，形成各类园区竞相发展的新态势。狠抓园区项目建设，节约、集约利用土地，提高土地产出效益。

扎实开展区域经济合作。以更加积极主动的姿态，全面加强与长三角和南京都市圈城市的联系与合作，主动对接，多边协作，互利共赢。积极借鉴沿海发达地区的成功经验，大胆实行“拿来主义”，构建符合市场经济规律的政策平台。深入推进与长三角城市的规划衔接，努力实现互动发展。充分利用沿江综合优势，大力拓展与皖江城市的合作领域，在沿江第一方阵东向发展中走在前列。

进一步扩大对外经济贸易。培育壮大外贸主体，优化出口商品结构，鼓励先进技术、关键设备和重要原材料进口。积极开展境外工程承包，促进技术和劳务输出，全年境外劳务输出300人以上。支持有条件的企业在境外建立销售网络和生产加工基地，拓展外向型经济发展空间。加强口岸建设，完善口岸功能，提升外贸服务水平。

（二）充分调动全社会积极性，大力推动全民创业

积极营造全民创业的浓厚氛围。创业是财富之源，只有全民创业，才能全民致富。充分调动广大人民群众和社会各界的积极性和创造性，把蕴藏在民间的创业潜能充分释放出来，把企业的市场主体地位充分显现出来，形成百姓创家业、能人创企业、干部创事业的生动局面。积极倡导“创业有功，致富光荣”的观念，把人民群众奔小康的强烈愿望转变成创业致富的强大动力，在全社会奏响奋力争先、勤劳致富的最强音。引导广大干部树立奋发有为的理念，鼓励探索，宽容失败，努力成为敢想敢干、敢冒风险、敢有作为的创新型人才。

全方位推动全民创业。广泛发动城乡群众自主创业，确保全年新增民营企业1000户以上。鼓励民营企业采取多种形式参与事业单位转企改制，围绕支柱产业和优势产业开展协作配套。发挥民营企业家生产管理有经验、市场开拓有门路、资本积累有基础的优势，当好创业表率，进行二次创业。支持工会、共青团、妇联等群团组织实施创新工程和创业行动，帮助困难群体、大中专毕业生、转业退伍军人在创业中谋发展。采取有效措施，吸引更多的外出务工能人返乡创业。鼓励农民创办经济实体，引导农民进城创业。

努力为全民创业提供优质高效服务。依托各类开发园区和工业集中区，发展各具特色的中小企业创业基地，构筑全民创业平台。充分利用社会职业教育资源，积极创新培训模式，加强对创业人员的创业培训，全年创业培训500人以上。面向失业人员、大中专毕业生及其他就业群体，开展灵活多样的创业指导，增强其创业能力和职业转换能力。进一步完善各类优惠政策整体联动的工作模式，加大对创业的政策支持力度。通过各方面共同努力，真正形成政府鼓励创业、社会支持创业、群众自主创业的良好发展环境。

（三）强势推进新型工业化，促进现代加工制造业加快发展

以大投入推动骨干企业优化升级。力争全年完成工业投资120亿元以上。支持马钢重点项目建设，建成车轮扩建项目，加快推进500万吨薄板带工程，启动实施40万吨硅钢片项目，确保2007年建成投产。突出抓好万能达二期扩建、山鹰造纸、雨润肉制品等一批重大项目建设，支持电厂、星马、圣戈班、中橡等企业加快扩建改造和产品升级，壮大骨干企业规模，增强工业发展优势。县区也要以加大投入为抓手，进一步做大做强工业，为全市工业发展提供有力支撑。

以优质服务促进中小企业加快发展。把2006年作为“企业服务年”，全面改善企业发展环境。建立中小企业服务中心，健全信息服务体系，提高服务水平。支持发展多层次、多类型的信用担保机构，新增担保机构5家以上，引导金融机构加大对中小企业的信贷扶持力度。争取天源科技公司上市融资，扎实做好方圆回转支承公司上市准备工作。鼓励和支持“专精特新”中小企业采取资本运营、外资嫁接等手段，扩大规模，提升档次，加快发展。积极推进磁性材料、博望机械加工制造、纺织服装等产业集群建设，推动中小企业特色化和集群化发展。

以新型工业化提升工业整体实力。积极开展以节能降耗为重点的技术改造，加快圣戈班大口径管件、方圆回转支承、天成高档面料等项目建设步伐，推动企业生产装备水平快速提升。深入推进国家“863”新材料产业园和成果转化基地建设，争创国家科创中心，加快科技成果产业化进程，促进高新技术产业加快发展。支持马钢等企业投资新材料产业，不断提升新材料产业发展规模和水平。鼓励企业管理创新，促进现代管理技术、信息技术在企业中的应用，提高企业管理现代化水平。加强企业认证体系建设，积极争创国家和省级名牌，提高企业市场竞争力。

（四）扎实做好“三农”工作，努力实现建设社会主义新农村良好开局

大力调整农业产业结构。按照“优质、高效、生态、安全”的要求，继续抓好苗木花卉、畜禽养殖、水产生态养殖、无公害蔬菜、优质粮油和经果林等优势农产品生产基地建设，促进农业规模化、品牌化、标准化和产业化发展。加快发展畜牧、水产、蔬菜、食用菌等主导产业，重点发展奶牛养殖和大规格河蟹生产，力争奶牛养殖规模达到1万头，大规格河蟹养殖比重扩大到35%。培育壮大骨干龙头企业，推动企业与基地、农户建立稳固的利益联结机制，力争新增年营销收入超2亿元企业1户、超亿元企业5户。扶持发展各类农民专业合作经济组织和农产品产业协会，提高农业生产组织化程度，增强抵御市场风险的能力。坚持把发展“三资”农业作为调整农业结构的重要举措，加大力度引导“三资”投入农业综合开发，确保全年引进农业项目资金4亿元以上，整体提升农业产业层次和发展水平，推进现代农业建设。

继续深化农村各项改革。认真落实中央和省市支持“三农”工作的政策措施，坚持“多予、少取、放活”，不断增强农业和农村经济发展活力。全面完成当涂县农村综合改革试点工作，提高“三农”社会化服务水平。按照依法、自愿、有偿的原则，在稳定和完善家庭承包经营制度的基础上，积极探索建立农村土地承包经营权流转新机制，推进农业适度规模经营。严格基本农田保护，加强土地整理，提高耕地质量，改善种植条件。积极推进农业技术推广体系改革，认真组织实施测土配方施肥、河蟹振兴、农业科技人员进村入户等工程，加快先进适用技术在农村经济发展中的示范和应用。

积极发展农村公共事业。围绕提高农业综合生产能力和改善农民生产生活条件，继续增加农业基础设施投入。续建新黄、澄湖公路，建成围乌公路和部省通达工程，完成“村村通水泥路”工程年度建设任务，争取黄池大桥项目立项。继续抓好堤防加固达标、涵闸水库除险改造和沿江泵站工程建设，提高城乡防汛抗旱排涝能力。以完善基础设施、拉开发展框架为重点，全年完成小城镇建设投资10亿元，开工建设3—5个农民集中居住示范区。加大农村教育扶持力度，调整农村中小学布局，推进农村学校标准化建设。继续完善农村公共卫生、文化、通讯等设施，加快实施20户以上的自然村“村村通有线电视”工程，提高农村公用设施保障水平。支持发展具有农村特点的循环经济，加强农村环境整治，清洁水源、清洁能源、清洁村庄，美化村容村貌。进一步完善新型农村合作医疗制度和特困家庭重病救助制度，加快推进农村安全饮水工程建设，扎实做好被征地农民的就业和社会保障工作。大力发展劳务经济，扎实推进“阳光工程”，全年新增劳务输出1.5万人以上。高度重视抓好高致病性禽流感等重大动物疫病和人畜共患疾病的防控，切实保障农业生产安全和农民生命健康。

（五）大力n lang="EN-US">

推动生产服务业加速发展。鼓励外来资本、民间资本进入生产服务业领域，促进生产服务业规模化发展。重点建设工业产品要素市场，加快形成钢材等工业产品的仓储、加工和配送体系。积极建设大型特色农产品批发市场和配套设施，逐步完善面向长三角的农产品销售网络。加快发展信息服务业，抓好信息网络基础设施建设，推进电子商务和社会服务领域信息化。进一步完善金融服务体系，加强金融网点建设，力争引进1—2家股份制商业银行来我市设立分支机构，支持证券、保险业加快发展。重视发展各类行业协会，积极发展咨询、律师、会计等中介服务业。

提升消费服务业规模。加快采石风景区、濮塘生态旅游度假区、小九华综合开发等旅游项目建设，积极开发郊区和当涂“农家乐”休闲旅游。广泛开展旅游促销活动，加强区域旅游合作，力争全年旅游业总收入超过14亿元。大力发展文化产业，繁荣文艺演出和艺术培训市场，促进印刷、出版发行等行业规范发展。全面实施“十一五”商业网点规划，建成大华购物中心一期工程、新都商品批发市场一期工程等大型商业网点，促进特色商业街发展。启动当涂县“全省农村商品流通改革和市场建设试点县”工作，完善农村商品流通网络体系。继续加大房地产开发力度，抓好廉租房和拆迁安置房建设，全年完成投资30亿元，竣工房屋面积180万平方米以上。完善住房消费服务体系，带动建材、装潢、家电、信息等相关产业发展。

进一步完善加快服务业发展的政策措施。坚持规划先行，出台实施《马鞍山市服务业跨越式发展（2006—2008）行动计划》。制定优惠政策，加大资金投入，重点支持商贸、科技、旅游、物流等产业发展壮大。促进服务业资源在市场上的合理流动，形成各类服务业充分竞争的格局。加大服务业人才引进和职业培训力度，为服务业发展提供强有力的人才和智力支撑。

（六）切实加强城市管理建设，实现市容市貌大改观

认真开展“城市管理年”活动。加强各类规划编制，严格规划执法和管理，切实发挥规划的龙头指导作用。加快完善“两级政府、三级管理、四级网络”的城市管理体系，形成市政府统一领导、城区政府具体实施、各部门齐抓共管、社会各界和市民群众积极参与的城市管理新体制。不断完善相对集中行政处罚权制度，加大城市管理执法力度，坚决依法拆除城市规划区内违法建设。继续实施危旧房改造、老旧小区整治，开展旧房出新工程??决好占道经营、道路扬尘等影响市容环境的重难点问题。进一步加强和改进土地征迁工作，及时满足重点项目用地需求。完成高污染燃料禁燃区炉窑整改工作，强化工业污染源的治理和监管，推动城市环境治理工作制度化、长效化。组织实施生态市建设规划，加快生态省综合示范基地项目建设步伐，加强矿区生态系统恢复和重建，按计划推进采石河综合整治，巩固提高创模成果。

进一步提升城市承载服务功能。全年完成城市建设投资50亿元以上。以慈湖地区路网建设为重点，新建联合路、曙光路，实施205国道改造二期工程，打通慈湖经济开发区和城市北部地区物流快速通道。延伸九华路、葛羊路、采秣路，贯通花园路，推动新建小区道路与城市主干道路对接，加快完善城市道路交通网络。建成王家山污水处理厂改扩建主体工程，完善城市南部道路污水管网，筹划建设东部污水处理厂，继续推进天然气利用工程，规划建设一批城市停车场，增强市政设施配套功能。加大港口码头建设力度，争取早日开工建设长江大桥和两端接线高速公路。加强城乡电网建设和改造，完善主网和配网结构，提高电网供电能力和供电可靠性。加快市政公园建设步伐，综合整治雨山湖公园、霍里山公园，提升城市园林绿化水平。

（七）深入推进重点领域改革，不断增强经济社会发展活力

加大力度推进行政管理体制改革。理顺市区两级管理体制，强化区级发展经济和管理社会的职能，进一步激发城区发展活力。建立健全与市、县（区）事权相匹配的财税体制，推进部门预算改革，完善重点支出和应急支出保障机制。实施县乡财政振兴工程，促进县域经济加快发展。深化乡镇机构改革，加快转变乡镇职能。深入推进水利工程管理体制改革，促进水资源的优化配置。加强政府性投资项目管理，强化对社会投资活动的引导、调控和监管。

积极稳妥推进事业单位改革。继续抓好生产经营类事业单位改革，全面完成转企改制任务，实现社会化投资、市场化运营、产业化发展。进一步理顺行政管理与社会服务的关系，积极做好行政执行类和公益类事业单位改革准备工作。以机构、产权、人事、分配等制度改革为重点，统筹推进事业单位综合配套改革试点工作。稳妥推进市政、园林、环卫体制改革，提高管理服务水平。积极推进文化体制改革，加强文化事业建设，大力发展文化产业，不断满足人民群众的精神文化需求。

巩固和扩大企业改革成果。妥善解决已改制企业遗留问题，保护职工合法权益，促进企业健康发展。优化改革方案，按时完成马钢、山鹰、星马等上市公司股权分置改革任务。积极探索加强国有资产监管的有效措施，健全监事会管理考核机制，强化对国有及国有控股企业经营管理者的业绩考核，建立和完善经营激励与约束机制。健全国有资产产权交易规则和监管制度，确保国有资产安全有效运营。

（八）统筹发展各项社会事业，积极构建和谐马鞍山

扎实做好就业和社会保障工作。把促进就业摆在经济社会发展更加突出的位置，加快城乡创业服务中心建设，逐步建立市场主导就业、政府促进就业、个人自谋职业相结合的长效机制。全年城镇新增就业2.1万人，其中失业人员再就业1.1万人，“4050”人员再就业2000人；实施职业技能培训1万人。继续完善多层次的社会保障体系，年末城镇职工养老、失业、医疗保险参保率达96%以上，工伤、生育保险参保率达75%。建立城镇非职工居民合作医疗保险制度，将城镇无业居民、灵活就业人员、大中小学学生及儿童逐步纳入医疗保障体系，积极探索外来务工人员医疗保障措施，在全省率先基本实现医疗保险全覆盖。进一步完善城乡居民最低生活保障制度，出台实施城市鳏寡孤独老人集中供养和城乡医疗救助政策。扎实开展“劳动保障执法监察年”活动，切实维护劳动者合法权益。继续集中力量办好10件左右为民办实事项目。

协调发展各项社会事业。全面推进义务教育均衡发展，加快普及高中阶段教育，推进职业教育现代化，鼓励发展民办职业教育。积极发展高等教育，继续支持安徽工业大学加快发展，力争在新城东区新办一所高校。创新办学体制，培育发展多种形式的民办教育。健全资助家庭困难学生就学制度，启动“关爱留守儿童”工程，切实保障困难家庭学生和流动人口子女受教育的权利。巩固扩大第一届中国诗歌节效应，营造更加浓厚的诗歌文化氛围。积极开展健康向上、群众喜闻乐见的文化活动，力争建成市博物馆、大剧院、图书馆主体工程，加大古文化遗址保护和发掘力度。探索建立市级卫生应急指挥信息平台，加快推进医疗卫生基础设施建设，大力发展社区卫生服务。加强对艾滋病、结核病、血吸虫病、狂犬病的防治，进一步提高对重大疾病的预防控制和医疗救治能力。深入开展全民健身运动，提高竞技体育水平。建设国家一级气象站，建立城乡公共气象、防震减灾服务体系。加大国防宣传和教育力度，继续稳妥做好军转干部和退伍士兵安置工作。加强人防设施建设，提高城市防空应急能力。积极推进档案管理模式改革，优化整合档案资源。推进广播电视数字化工程，认真做好第二次农业普查工作，积极开发利用第一次经济普查成果，完成《马鞍山市志》续志，加强软件应用管理，统筹发展其他社会事业。继续抓好文明城市创建工作。大力推进青少年活动阵地建设，突出抓好未成年人思想道德建设和大学生思想政治教育。加快联合征信网络平台建设，初步建立企业和涉信人群信用信息数据库，启动信用评价及发布系统。扎实开展“双拥”工作，力争进入“全国双拥模范城市”行列。完善社区管理体制，强化小区物业管理，增强服务居民的能力和水平。

深入推进“平安马鞍山”建设。完善和创新社会治安防控体系，建设城市报警和监控技术系统，大力整治校园周边、公共娱乐场所、居民小区治安秩序，严厉打击各种刑事犯罪活动，切实增强人民群众的安全感。进一步加强危机管理，积极预防、妥善处置群体性事件。正确处理新时期的人民内部矛盾，完善信访工作责任制，健全社会利益协调和矛盾纠纷调处机制。认真落实安全生产责任制，严防重大安全事故发生，巩固和发展安定团结的政治局面。

（九）努力提高政府管理服务水平，建设人民满意政府

积极改进行政管理方式。加快转变政府职能，推进政企、政资、政事分开，更好地发挥政府经济调节、市场监管、社会管理、公共服务的职能。坚持依法行政，深入贯彻行政许可法，进一步减少和规范行政许可。继续加强和改善行政执法，严格行政执法责任制度和过错追究制度，提高行政执法水平。加强对涉及经济社会发展全局的战略性、前瞻性问题的调查研究，健全重大事项决策的协商和协调机制、专家咨询制度、社会公示和听证制度，推进决策的科学化、民主化。

着力强化行政效能建设。出台落实提高行政领导工作效能的具体规定，建立健全和严格执行效能问责制，切实提高政府机关特别是行政领导的工作效能。进一步完善和强化目标责任管理，加大考核奖惩力度，完善体现科学发展观和正确政绩观要求的干部实绩考核评价制度。加强对重大决策和重要工作部署的跟踪督查，深入推进效能监察，坚决改变极少数部门和工作人员推诿扯皮、办事效率不高的现象，提高政府执行力和公信力。主动接受市人大的法律监督和工作监督，接受市政协的民主监督，认真办好人大代表议案、建议和政协提案，广泛听取各民主党派、工商联、无党派人士和各人民团体的意见。继续办好“政风行风”热线，自觉接受群众和社会舆论的监督，不断改进政府工作。

全面优化服务环境。政府系统全体工作人员要进一步解放思想、更新观念，坚决冲破一切影响发展的思想束缚和体制障碍，牢固树立服务经济、发展为上、改革创新的理念，以思想大解放促进经济社会大发展。要始终保持奋发有为、奋力争先的精神状态和求真务实的工作作风，勤于思考，勇于开拓，敢于负责，快速落实，不断开创各项工作新局面。要把思想解放落到实处，紧紧围绕招商引资、全民创业等重点工作，千方百计为市场主体创造公平竞争环境，提供优质高效服务。要继续完善行政服务中心功能，加快电子政务建设步伐，积极推进政务公开。要以贯彻实施《公务员法》为契机，加强对公务员的教育、培训和管理，不断提高工作效率和服务水平。要认真贯彻中央和省、市惩治和预防腐败的有关规定，教育广大公务员特别是领导干部自觉廉洁从政，树立政府良好形象。

各位代表，今年是马鞍山建市五十周年。回首往昔，我们豪情满怀；展望未来，我们信心倍增。让我们紧密团结在以胡锦涛同志为总书记的党中央周围，在省委省政府和市委的坚强领导下，依靠和组织全市人民，埋头苦干，奋发进取，出色完成今年各项目标任务，以优异成绩向建市五十周年献上一份厚礼，为加速实现“两个率先”、努力构建和谐社会而不懈奋斗！
